# Sleep and dementia: Assessing established dementia‐related factors using multivariable Mendelian randomization

**DOI:** 10.1002/alz.71592

**Published:** 2026-06-15

**Authors:** Yunyun Guo, Rui Wang, Shireen Sindi, Lefkos T Middleton, Miia Kivipelto

**Affiliations:** ^1^ The Ageing Epidemiology (AGE) Research Unit School of Public Health Imperial College London London UK; ^2^ Institute of Science and Technology for Brain‐Inspired Intelligence (ISTBI) School of Public Health Fudan University Shanghai China; ^3^ Division of Clinical Geriatrics Department of Neurobiology Care Sciences and Society, Karolinska Institute, Center for Alzheimer Research QA32 Solna Sweden; ^4^ Department of Health Sciences the Swedish School of Sport and Health Sciences Stockholm Sweden; ^5^ Wisconsin Alzheimer's Disease Research Center, School of Medicine and Public Health University of Wisconsin Madison Wisconsin USA; ^6^ Public Health Directorate Imperial College NHS Healthcare Trust London UK; ^7^ Department of Neurology, Institute of Clinical Medicine University of Eastern Finland Kuopio Finland; ^8^ Theme Inflammation and Aging Karolinska University Hospital, Stockholm Solna Sweden; ^9^ Institute of Public Health and Clinical Nutrition University of Eastern Finland Kuopio Finland

**Keywords:** Causal inference, dementia, Mendelian randomization, sleepiness

## Abstract

**INTRODUCTION:**

The relationship between sleep, other dementia risk factors, and dementia remains unclear. We aim to explore these associations using a two‐sample Mendelian randomization (MR) approach.

**METHODS:**

We identified genetic instrumental variables for sleep characteristics from the UK Biobank and obtained summary‐level genetic association data for dementia outcomes, including Alzheimer’s disease (AD), from the FinnGen consortium. We applied inverse‐variance weighted (IVW) MR to examine the associations between sleep characteristics and dementia outcomes. Multivariable MR was subsequently employed to assess the influence of additional dementia‐relevant factors on these relationships.

**RESULTS:**

Genetic variants for insomnia, among the sleep characteristics, were associated with elevated AD risk (odds ratio [OR]_IVW _= 3.02, 95% confidence interval [CI]: 1.28–7.10, *p *= 0.01). Yet this association was largely attenuated after adjusting for education and low‐density lipoprotein (LDL).

**DISCUSSION:**

These findings indicate that the association between insomnia and AD is complex and indirect, potentially operating through social‐behavioral factors (education) and biological pathways such as LDL cholesterol.

## Highlights

- Genetic variants associated with insomnia are linked to an increased risk of Alzheimer's disease. This relationship appears to be largely modified or mediated by genetic variants related to education and low‐density lipoprotein levels.
- Genetic liability to dementia was initially associated with lower insomnia risk in reverse Mendelian randomization (MR) analyses, but this association was attenuated after excluding apolipoprotein E (*APOE*) region variants.

## 1 BACKGROUND

As the global population ages rapidly, neurodegenerative diseases pose an increasing public health challenge, specifically, Alzheimer's disease (AD) and other late‐onset dementia.^1^, ^2^ The latest Lancet Dementia Commission report summarized 14 modifiable lifelong health and lifestyle factors for dementia and suggested that nearly half of all dementia cases could be prevented by targeting these factors.^3^ Beyond established modifiable risk factors, sleep characteristics are increasingly recognized as influential in dementia outcomes,^3^ partly due to pathophysiological mechanisms such as impaired clearance and accumulation of amyloid beta (Aβ) and hyperphosphorylated tau (p‐tau) during sleep deprivation or fragmented sleep.^4^, ^5^, ^6^ In addition, sleep disturbances are linked to cerebrovascular injury and blood–brain barrier dysfunction, which can lead to neuronal damage and cognitive decline.^4^, ^5^, ^6^

Evidence from observational studies has directed that dementia risk may link to certain sleep characteristics, such as daytime napping, daytime sleepiness, sleep duration, insomnia, and chronotype.^7^, ^8^, ^9^, ^10^ However, substantial inconsistency remains across these studies, particularly when examining more specific dementia outcomes.^7^, ^8^, ^9^, ^10^ Heterogeneity across studies may largely be explained by differences in sleep definitions, baseline age, confounder adjustment, and limited control for potential reverse causality. Although RCTs are needed to further verify the sleep–dementia relationship, they are often difficult to conduct due to long follow‐up, complex causation, and logistical challenges.^11^ Under such circumstances, Mendelian randomization (MR) provides an approach that leverages the principles of random allocation, using genetic variants as instrumental variables (IVs) to test causal hypotheses that are difficult to evaluate in observational studies. These genetic variants are assigned randomly at conception and serve as natural proxies that help minimize confounding and reduce the risk of reverse causality.^11^ Despite growing interest in the relationship between sleep and dementia, MR studies examining this association remain limited and warrant further investigation.^12^

Previous MR studies have investigated the potential causal role of sleep characteristics in cognitive outcomes and dementia, but the findings have been mixed ^13^, ^14^, ^15^, ^16^, ^17^, ^18^, ^19^, ^20^, ^21^, ^22^, ^23^, ^24^, ^25^, ^26^ (see Table 1 and Table S1). An overview of key findings from the literature, six studies has reported a causal effect of sleep duration, daytime napping, and insomnia on cognitive performance (reaction time) and AD,^13^, ^15^, ^16^, ^18^, ^21^, ^26^ while evidence from other studies have yielded inconclusive findings, showing null, weak associations.^14^, ^17^, ^19^, ^20^, ^22^, ^23^, ^24^, ^25^ Several methodological limitations and research gaps need to be acknowledged in interpreting these MR results. First, few studies conducted comprehensive sensitivity analyses, particularly regarding horizontal pleiotropy, to assess potential biases arising from violations of MR assumptions.^14^, ^25^, ^26^ In addition, sample overlap between the sleep and dementia datasets (15) may inflate the Type I error rate and bias estimated causal effects. Second, most studies relied on univariable MR analyses, with multivariate MR (MVMR) approaches,^15^, ^18^, ^21^ focusing primarily on sleep characteristics.^13^, ^14^, ^15^, ^16^, ^17^, ^18^, ^19^, ^20^, ^21^, ^22^, ^23^, ^24^, ^25^, ^26^ To date, no study has examined the potential impact of other established dementia risk factors, such as the modifiable risk factors identified by the Lancet Commission Report. Finally, although much of the existing evidence has focused on all‐cause dementia and AD, data on other cause‐specific dementia remain scarce.

**TABLE 1 alz71592-tbl-0001:** Summary of previously published two‐sample Mendelian randomization studies on instrumental variables of sleep and dementia outcomes.

Study	Exposure	Outcome	Study design	*N* (outcome)	Sensitivity analyses	MVMR	OR/β (95% CI)	*p*‐value
Guo, 2025 ^13^	Short sleep duration	Alzheimer's disease	Two‐ sample MR	218,792	Limited (Pleiotropy: MR‐Egger intercept; Heterogeneity: Cochran's Q)	No	1.00 (0.74 to 1.35)	0.980
	Long sleep duration	Alzheimer's disease	Two‐ sample MR	218,792			1.20 (0.82 to 1.76)	0.340
	Insomnia	Alzheimer's disease	Two sample MR	218,792			1.13 (1.02 to 1.24)	0.020
Guo, Harshfield and Markus, 2024 ^14^	Sleep duration	Alzheimer's disease	Two‐ sample MR	21,982	No	No	0.841 (0.573 to 1.236)	0.378
	Insomnia	Alzheimer's disease	Two‐ sample MR	21,982			0.981 (0.685 to 1.407)	0.919
	Chronotype	Alzheimer's disease	Two‐ sample MR	21,982			0.994 (0.798 to 1.238)	0.956
	Napping	Alzheimer's disease	Two‐ sample MR	21,982			0.767 (0.453 to 1.299)	0.324
	Daytime dozing	Alzheimer's disease	Two‐ sample MR	21,982			1.105 (0.448 to 2.727)	0.829
	Snoring	Alzheimer's disease	Two‐ sample MR	21,982			0.938 (0.284 to 3.101)	0.917
Xiang et al., 2024 ^15^	Insomnia	Alzheimer's disease	Two‐ sample MR	488,285	Limited (Heterogeneity: Funnel plot, Cochran's Q; Pleiotropy: MR Egger intercept, MR‐PRESSO, Leave‐one‐out analysis)	In a multivariate MR study, seven sleep characteristics corrected for each other.	1.000 (0.997 to 1.003)	0.766
	Sleep duration	Alzheimer's disease	Two‐ sample MR	488,285			1.002 (1.000 to 1.004)	0.046
	Nap during day	Alzheimer's disease	Two‐ sample MR	488,285			0.999 (0.997 to 1.002)	0.605
	Chronotype	Alzheimer's disease	Two‐ sample MR	488,285	1.000 (0.999 to 1.001)	0.779
	Daytime dozing	Alzheimer's disease	Two‐ sample MR	488,285			1.001 (0.996 to 1.006)	0.591
	Snoring	Alzheimer's disease	Two‐ sample MR	488,285			1.001 (0.996 to 1.005)	0.721
	Getting up in morning	Alzheimer's disease	Two‐ sample MR	488,285			0.999 (0.997 to 1.001)	0.405
Ran et al., 2024 ^16^	Daytime napping	Alzheimer's Disease	Two‐ sample MR	1489	Limited (Pleiotropy: MR‐Egger intercept; Heterogeneity: Cochran's Q)	No	75.62 (4.64 to 1232.59)	0.002
	Daytime napping	Family history of Alzheimer's disease	Two‐ sample MR	408,942			1.06 (1.01 to 1.11)	0.010
	Daytime napping	Dementia, including AvoHilmo	Two‐ sample MR	218,792			3.14 (1.04 to 9.48)	0.042
	Alzheimer Disease	Daytime napping	Two‐ sample MR	462,400			0.993 (0.990 to 0.997)	< 0.001
	Family history of Alzheimer's disease	Daytime napping	Two‐ sample MR	462,400			0.88 (0.83 to 0.94)	< 0.001
	Dementia with Lewy bodies	Daytime napping	Two‐ sample MR	462,400			0.995 (0.990 to 0.999)	0.012
	Any dementia	Daytime napping	Two‐ sample MR	462,400			0.993 (0.990 to 0.997)	<0.001
Xiong et al., 2024 ^17^	Short sleep duration	All‐cause dementia	Two‐ sample MR	7223	Limited (Pleiotropy: MR‐Egger intercept; Heterogeneity: Cochran's Q)	No	0.40 (0.13 to 1.28)	0.120
	Long sleep duration	All‐cause dementia	Two‐ sample MR	7223			2.54 (0.09 to 72.30)	0.590
	Short sleep duration	Alzheimer's Disease	Two‐ sample MR	6930			1.15 (0.47 to 2.83)	0.760
	Long sleep duration	Alzheimer Disease	Two‐ sample MR	6930			2.08 (0.34 to 12.68)	0.430
Grover and Sharma, 2022 ^18^	Sleep duration	Alzheimer's disease	Two‐ sample MR	33,976	Limited (MR‐Egger, weighted median and weighted mode; Leave‐one‐out analysis; Scatter plots and Funnel plots;)	In a multivariate MR study, this study adjusted for quantity of sleep, sleep preference for a given time of day, and pain.	0.992 (0.956 to 1.029)	0.657
	Short sleep	Alzheimer's disease	Two‐ sample MR	33,976			1.256 (1.081 to 1.459)	0.004
	Long sleep	Alzheimer's disease	Two‐ sample MR	33,976			0.877 (0.527 to 1.460)	0.538
	Chronotype	Alzheimer's disease	Two‐ sample MR	33,976			0.995 (0.973 to 1.018)	0.673
	Morning person	Alzheimer's disease	Two‐ sample MR	33,976			1.001 (0.986 to 1.017)	0.844
	Insomnia	Alzheimer's disease	Two‐ sample MR	33,976			0.981 (0.939 to 1.024)	0.345
Yuan et al., 2022 ^19^	Sleep duration	Alzheimer's disease	Two‐ sample MR	63,926	Limited (Pleiotropy: MR‐Egger intercept; Heterogeneity: Cochran's Q)	No	0.856 (0.498 to 1.473)	0.575
Li et al., 2022 ^20^	Daytime napping	Alzheimer's disease	Two‐ sample MR	63,926	Limited (Pleiotropy: MR‐Egger intercept test and MR‐PRESSO)	No	0.76 (0.53 to 1.10)	0.140
	Alzheimer Disease	Daytime napping	Two‐ sample MR	452,633			β: −0.006 (−0.009 to −0.002)	0.002
Chen et al., 2022 ^21^	Insomnia	Alzheimer's disease	Two‐ sample MR	455,258	Limited (Pleiotropy: weighted median, simple median, MR‐RAPS, MR‐Egger, and MR‐PRESSO)	In a multivariate MR study, this study adjusted for sleep duration and major depression.	β:0.021 (0.011 to 0.031)	< 0.001
	Sleep duration	Alzheimer's disease	Two‐ sample MR	455,258			β: −0.049 (−0.094 to −0.004)	0.036
Andrews et al., 2021 ^22^	Insomnia	Late‐onset Alzheimer's disease	Two‐ sample MR	54,162	Limited (Heterogeneity: Cochran's Q; Pleiotropy: MR Egger intercept)	No	β: −0.025 (−0.456 to 0.406)	0.910
	Sleep duration	Late‐onset Alzheimer's disease	Two‐ sample MR	54,162			β: −0.035 (−0.270 to 0.200)	0.780
Cullell et al., 2021 ^23^	Sleep efficiency	Alzheimer's disease	Two‐ sample MR	21,235	Limited (MR‐Egger, weighted median; Heterogeneity: Cochran's Q; Pleiotropy: MR Egger intercept, MR‐PRESSO, Leave‐one‐out analysis)	No	β: −10.53 (−20.389 to −0.671)	0.036
	Chronotype	Alzheimer's disease	Two‐ sample MR	21,235			Not available	0.088
	Insomnia	Alzheimer's disease	Two‐ sample MR	21,235			Not available	0.593
	Sleep duration	Alzheimer's disease	Two‐ sample MR	21,235			Not available	0.443
	Daytime Sleepiness	Alzheimer's disease	Two‐ sample MR	21,235			Not available	0.524
Anderson et al., 2020 ^24^	Chronotype	Alzheimer's disease	Two‐ sample MR	54,162	Limited (Weighted median and MR‐Egger regression; Heterogeneity: Funnel plot, Cochran's Q; Pleiotropy: Leave‐one‐out analysis)	No	1.02 (0.90 to 1.16)	0.770
	Sleep duration	Alzheimer's disease	Two‐ sample MR	54,162			0.94 (0.71 to 1.25)	0.660
	Sleep fragmentation	Alzheimer's disease	Two‐ sample MR	54,162			1.12 (0.87 to 1.45)	0.370
	Insomnia	Alzheimer's disease	Two‐ sample MR	54,162			1.00 (0.59 to 1.68)	0.990
	Daytime napping	Alzheimer's disease	Two‐ sample MR	54,162			0.67 (0.45 to 1.02)	0.060
	Daytime sleepiness	Alzheimer's disease	Two‐ sample MR	54,162			0.65 (0.29 to 1.43)	0.280
Huang et al., 2020 ^25^	Chronotype	Alzheimer's disease	Two‐ sample MR	63,926	Limited (Weighted median and MR‐Egger regression; MR‐PRESSO)	No	0.992 (0.912 to 1.079)	0.851
	Insomnia	Alzheimer's disease	Two‐ sample MR	63,926			0.994 (0.638 to 1.550)	0.980
	Self‐reported sleep duration	Alzheimer's disease	Two‐ sample MR	63,926			0.943 (0.748 to 1.190)	0.622
	Number of sleep episodes	Alzheimer's disease	Two‐ sample MR	63,926			0.770 (0.606 to 0.979)	0.033
	Least active 5 hours [L5] timing	Alzheimer's disease	Two‐ sample MR	63,926			1.063 (0.704 to 1.604)	0.773
	Sleep efficiency	Alzheimer's disease	Two‐ sample MR	63,926			1.282 (0.799 to 2.059)	0.302
	Alzheimer's disease	Chronotype	Two‐ sample MR	403,195			1.01 (1.01–1.02)	0.001
	Alzheimer's disease	Insomnia	Two‐ sample MR	237,627	0.99 (0.991–0.995)	< 0.001
	Alzheimer's disease	Self‐reported sleep duration	Two‐ sample MR	446,118			β: −0.006 (−0.010 to −0.002)	< 0.001
	Alzheimer's disease	Number of sleep episodes	Two‐ sample MR	84,441			β: ‐0.025 (−0.031 to −0.019)	< 0.001
	Alzheimer's disease	Least active 5 hours [L5] timing	Two‐ sample MR	85,205			β: −0.024 (−0.030 to −0.018)	< 0.001
	Alzheimer's disease	Sleep efficiency	Two‐ sample MR	84,810			β: 0.003 (−0.006 to 0.007)	0.901
Henry et al., 2019 ^26^	Sleep duration	Visual memory	Split‐sample MR	395,803	Limited (Weighted median; Pleiotropy: MR Egger intercept)	No	1.028 (0.993 to 1.063)	0.115
	Sleep duration	Reaction time	Split‐sample MR	395,803			1.011 (1.002 to 1.021)	0.023
	Sleep duration	All‐cause dementia	Split‐sample MR	311,903			1.183 (0.641 to 2.182)	0.591
	Sleep duration	Alzheimer's disease	Two‐ sample MR	54,162			0.886 (0.665 to 1.180)	0.408

*Notes*: Studies included in this table were identified through a PubMed search conducted from database inception to April 27, 2026 using the following strategy: (((((((sleep) OR (insomnia)) OR (napping)) OR (sleepiness)) OR (morning person)) OR (chronotype)) AND (((dementia) OR (Alzheimer's disease)) OR (vascular dementia))) AND (Mendelian randomization). Of the 63 studies screened, 14 were included after excluding duplicate records, systematic reviews, studies not using MR methods, and studies that did not examine the sleep traits or dementia outcomes of interest. Detailed reasons for exclusion are provided in Table S1. This table is intended as a non‐systematic summary of selected studies rather than a formal systematic review.

Abbreviations: CI, confidence interval; MR, Mendelian randomization; MR‐PRESSO, Mendelian Randomization Pleiotropy RESidual Sum and Outlier; MR‐RAPS, Mendelian randomization robust adjusted profile score; MVMR, multivariable Mendelian randomization; OR, odds ratio.

This study aimed to systematically assess the relationships between sleep characteristics (i.e., daytime napping, daytime sleepiness, sleep duration, insomnia, and chronotype) and dementia outcomes using a two‐sample MR design. Furthermore, we sought to investigate whether other established dementia risk factors occurring in early‐life and midlife influence the relationship between sleep characteristics and dementia.

## 2 METHODS

### 2.1 MR study design

Study populations were based mainly on the UK Biobank and the FinnGen dataset.^27^, ^28^ The data used in this study were obtained from large genome‐wide association studies (GWASs), and the original cohorts included appropriate ethical clearance approvals and patient informed consent instructions. Therefore, no separate ethical approval was required for this study. We applied two‐sample MR analyses in this study. Based on the UK Biobank, we derived genetic instruments for five specific sleep characteristics (Sample 1),^27^ whereas the dementia outcome‐related genetic variants were derived from the FinnGen population (Sample 2).^28^

**RESEARCH IN CONTEXT**

**Systematic review**: Previous observational studies linking sleep characteristics to dementia have been limited by residual confounding and reverse causation. Mendelian randomization (MR), which mimics the randomization of randomized controlled trials (RCTs) at the genetic level, offers a less biased approach. However, prior MR studies on sleep and dementia have yielded inconsistent findings. Many lacked sufficient sensitivity analyses to assess horizontal pleiotropy, raising concerns about bias. Most used univariate MR, and even multivariate approaches, rarely accounted for broader dementia risk factors. Notably, none have adjusted for the modifiable risk factors highlighted by the Lancet Commission. Research has also largely focused on all‐cause dementia and Alzheimer's disease (AD), with limited evidence for other dementia subtypes. In addition, sample overlap in some studies may have inflated Type I error rates and biased estimates.

**Interpretation**: We applied multivariate MR to examine potential causal links between sleep characteristics and dementia. Using sleep‐related genetic variants as instruments, univariate MR revealed a significant association between insomnia and AD. However, this association was substantially attenuated after adjusting for genetic variants related to education and LDL cholesterol in multivariate MR. In the reverse MR analyses, we initially observed that genetic liability to all‐cause dementia and AD was associated with a lower likelihood of insomnia. However, these associations were attenuated and no longer statistically significant after exclusion of apolipoprotein E (*APOE*)‐region variants, suggesting that the observed reverse‐direction signals may have been largely driven by the *APOE* locus.

**Future directions**: Future studies should incorporate more diverse genome‐wide association study (GWAS) datasets to strengthen instrument validity, particularly for specific dementia subtypes. Integrating longitudinal data and investigating underlying mechanisms may help clarify the complex relationships between sleep, education, low‐density lipoprotein (LDL), and AD.

**FIGURE 1 alz71592-fig-0001:**
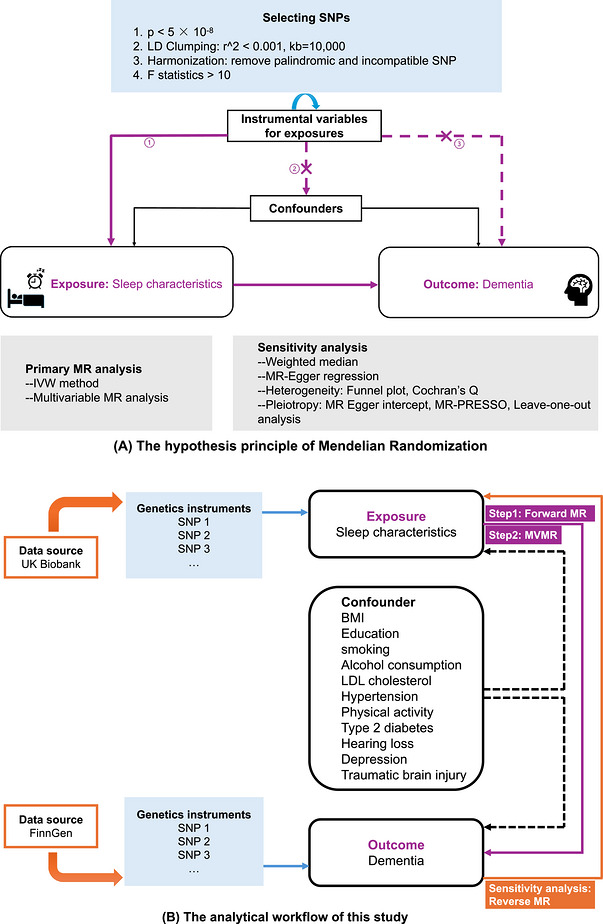
(A) Graphical representation of the core assumptions of the Mendelian randomization (MR) study and the analytical steps involved. Notes. A displays three hypothesis of MR. First, the genetic variant must be strongly associated with the exposure variable (self‐reported sleep phenotypes or dementia in separate pathways). Second, it should be unrelated to potential confounders. Third, it must not have a direct effect on the outcome—dementia in the main path (marked in purple) and sleep phenotypes in the reverse path (marked in orange)—but should influence the outcome only through pathways involving the exposure (sleep phenotypes in the main path and dementia in the reverse path). (B) Illustrates the analytical workflow. In Step 1, we performed MR to examine the relationship between genetic variants associated with sleep characteristics and dementia risk. In Step 2, we conducted multivariable Mendelian randomization (MVMR) to assess whether genetic instruments for other potential dementia risk factors occurring earlier in life influence the relationship between sleep phenotypes and dementia. Each factor was introduced into the model one by one. Finally, in sensitivity analysis, we tested the reverse association by using genetic instruments for dementia as the exposure and linking them to different sleep phenotypes as outcomes. Abbreviations. BMI, body mass index, LDL, low‐density lipoprotein; IVW, inverse‐variance weighted; kb, kilobase; LD, linkage disequilibrium; MR, Mendelian randomization; MR‐Egger, Mendelian Randomization‐Egger; MR‐PRESSO, Mendelian Randomization Pleiotropy RESidual Sum and Outlier; MWMR, multivariable Mendelian randomization; SNP, single nucleotide polymorphism

Figure 1 demonstrated detailed information on the core assumptions of the MR analyses and analytical workflow. Briefly, our analyses considered the three key assumptions of MR—relevance, independence, and exclusion restriction. The relevance assumption requires that the genetic variants used as IVs are strongly associated with the exposure. Independence assumes that IVs are not related to potential confounders that could bias the exposure–outcome relationship. The exclusion restriction assumption states that IVs should influence the outcome only through the exposure and not via alternative pathways. We conducted MVMR analyses to assess whether other potential dementia risk factors from early‐life or midlife influence the relationship between genetically proxied sleep characteristics and dementia.

### 2.2 Data sources and instruments

#### 2.2.1 Sleep characteristics

All genetic variants of sleep characteristics[Table alz71592-tbl-0001] were identified in GWASs at the UK Biobank. Based on evidence from previous observational studies and systematic reviews,^7^, ^8^, ^9^, ^10^ as well as data available in the UK Biobank, we included five self‐reported sleep characteristics in the present study: daytime napping, daytime sleepiness, sleep duration, insomnia, and chronotype. Daytime napping was defined as three categories: never or less than once per week, 1–3 times per week, or ≥4 times per week.^29^ Daytime sleepiness was categorized as never, sometimes, often, or all the time. Daytime napping and daytime sleepiness were treated as categorically ordered variables in the GWAS analyses.^30^ Sleep duration was defined as the total estimated sleep hours during a regular day (including any naps) ^31^ and treated as a continuous variable in the GWAS analyses. Insomnia was identified when a participant reported frequent difficulty falling asleep or waking up in the middle of the night.^32^ A chronotype (morningness preference) was identified when a participant responded with “Definitely a morning person” or “More of a morning than an evening person”.^33^ All selected single‐nucleotide polymorphisms (SNPs) related to sleep characteristics were based on the GWAS conducted on European populations.^29^, ^30^, ^31^, ^32^, ^33^ Detailed information on sleep characteristic—related SNPs is provided in Table S2.

#### 2.2.2 Incident dementia

We included all‐cause dementia, AD, and vascular dementia (VaD) in the main MR analyses. Genetic data for all‐cause dementia, AD, and VaD were obtained from the FinnGen project, which in 2021, conducted genetic association analysis of these three dementia‐related phenotypes in European populations.^28^ FinnGen was selected as the source of dementia outcome data to minimize potential sample overlap with the UK Biobank–based GWAS used for the sleep‐related exposures. In two‐sample MR, sample overlap can reduce the independence of SNP–exposure and SNP–outcome estimates, increase weak‐instrument bias, and potentially inflate Type I error.^11^ We therefore considered the use of an independent outcome dataset a conservative design choice. The sample sizes varied, from 212,159 participants for VaD to 218,792 participants for AD. All phenotypes were analyzed as binary traits, with the number of SNPs used ranging from 16,380,457 for VaD to 16,380,466 for AD. The corresponding GWAS IDs were shown in Table S2.

#### 2.2.3 MVMR included early‐life and midlife dementia risk factors

In line with the Lancet Commission, we included educational attainment (years of schooling) as an early‐life dementia risk factor, and the following as midlife risk/protective factors: hearing loss, low‐density lipoprotein (LDL) cholesterol level, depression, traumatic brain injury, physical activity, type 2 diabetes, smoking, hypertension, body mass index (BMI), and alcohol consumption. GWAS information is shown in Table S2.

### 2.3 Selection of genetic instruments

All SNPs reaching genome‐wide significance (*P* < 5 × 10^−8^) were selected as IVs and pruned for independence using an *r* ^2^ < 0.001 and a 10,000 kb clumping window. Data from sleep characteristics and dementia GWASs were harmonized to align effect alleles, with palindromic SNPs (A/T or G/C) excluded to avoid strand ambiguity. Only SNPs with[Fig alz71592-fig-0001] consistent strand orientation were retained. To minimize confounding, we further screened all selected SNPs in the PhenoScanner database to identify and exclude variants associated with potential cognitive‐related traits. In addition, for each MVMR model, a combined candidate SNP set was constructed from the included exposures, representing the union of instruments across those exposures. The corresponding SNP–exposure association estimates were then extracted across insomnia and each covariate. Effect estimates were harmonized so that the same effect allele was aligned across the exposure GWAS datasets for each SNP. The corresponding SNP–outcome associations were subsequently extracted and harmonized with the exposure data to ensure consistent effect allele orientation between exposures and outcomes. This harmonization process included strand alignment and the handling of palindromic variants. Effect allele frequencies were used, where available, to infer strand orientation. Palindromic SNPs with ambiguous strand orientation, particularly those with intermediate allele frequencies, were excluded. In the outcome datasets, SNPs absent from a given dementia GWAS were excluded rather than replaced by proxy SNPs. The final MVMR models were based on a common harmonized SNP set across exposures. F‐statistics were calculated to assess instrument strength, with values >10 indicating a low risk of weak instrument bias. The SNP selection workflow is illustrated in Figure S1, and the number of instruments for insomnia and each covariate's phenotype, together with the final number of SNPs included in each model, is shown in Table S3.

### 2.4 Statistical analysis

#### 2.4.1 Univariable MR between sleep characteristics and dementia

We conducted univariable MR to estimate the causal effects of five sleep characteristics on all‐cause dementia, AD, and VaD. SNPs strongly associated with each exposure were selected as IVs, and the inverse‐variance weighted (IVW) method was used to combine SNP‐specific estimates, giving more weight to precise instruments.^34^ For exposures with only one IV, the Wald ratio test was applied.^35^ The IVW analysis assumes that all IVs affect dementia only through the sleep characteristics.

#### 2.4.2 Multivariable MR analysis

Based on the results from the univariable MR, we further performed MVMR to separately estimate the direct effect of sleep characteristics on dementia outcomes, conditional on years of education, hearing loss, LDL cholesterol level, depression, traumatic brain injury, physical activity, type 2 diabetes, smoking, hypertension, BMI, and frequency of alcohol consumption.^36^ We again used IVW as the main method.

#### 2.4.3 Sensitivity and additional analyses

To assess robustness of MR analyses, five sensitivity analyses were performed. ^1^ MR‐Egger regression and the weighted median estimator (WME) were used alongside IVW. These methods relax assumptions about horizontal pleiotropy and increase result reliability.^2^, ^37^, ^38^ A funnel plot and Cochran's Q test assessed heterogeneity.^39^, ^40^ When heterogeneity was detected, a random‐effects IVW model was applied.^3^ The MR‐Egger intercept tested for horizontal pleiotropy (*p* > 0.05, indicating no pleiotropy).^4^, ^41^ The MR Pleiotropy RESidual Sum and Outlier (MR‐PRESSO) test was used to detect and remove pleiotropic outliers, followed by re‐analysis.^5^, ^42^ Leave‐one‐out analyses evaluated the influence of individual SNPs. We used MR‐Egger‐intercept tests to test horizontal pleiotropy in MVMR analyses.^43^, ^44^

To enhance the rigor of the results, the Bonferroni correction was applied to delineate the significance threshold (0.05/20 MR estimates = 0.0025). Results with *p* < 0.0025 were considered statistically significant, whereas results with 0.0025 <* p* < 0.05 were deemed suggestive, indicating they did not meet the Bonferroni‐corrected significance level but still reached the traditional 0.05 significance level.

In addition to the main MR analysis, we conducted additional analyses including other cause‐specific dementias, such as early‐onset AD, late‐onset AD, dementia with Lewy bodies (DLB), Parkinson's disease dementia (PDD), and Parkinson's disease. A power analysis was conducted in response to the wide confidence intervals (CIs) observed. We additionally conducted bidirectional univariable MR analyses to address reverse causality between sleep characteristics and dementia outcomes. Genetic variants for dementia and subtypes were used as IVs, applying the same IV selection and MR methods (IVW, MR‐Egger, WME) for consistency. For the reverse‐direction analyses, we further conducted a sensitivity analysis excluding variants located within ± 250 kb of the apolipoprotein E (*APOE*) ε4–defining SNP, rs429358, to assess whether the observed associations were driven by the *APOE* region.

All statistical analyses were conducted using the TwoSampleMR package (version 0.5.7) implemented in R software (version 4.2.2; R Foundation for Statistical Computing, Vienna, Austria).^45^, ^46^

## 3 RESULTS

### 3.1 Instrumental variable selection

The F‐statistics for the association between IVs and sleep characteristics ranged from 42.47 to 46.54 (Table S4), indicating a low risk of weak instrument bias in this study. Additional screening was conducted based on IV selection criteria, with results presented in Tables S5–S9. After quality control and screening, a total of 102 SNPs were included as genetic instruments for daytime napping, 31 SNPs for daytime sleepiness, 74 SNPs for sleep duration, 44 SNPs for insomnia, and 138 SNPs for chronotype.

### 3.2 Univariable MR between sleep characteristics and dementia

**FIGURE 2:**
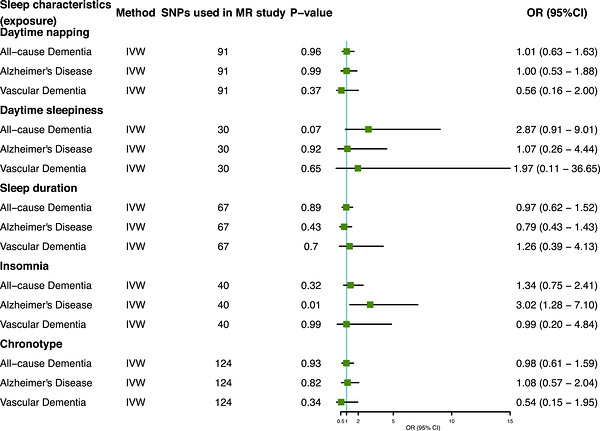
Forest plot of Mendelian randomization (MR) analyses for the relevance of sleep phenotypes with risk of dementia. Abbreviations. OR, odds ratio; CI, confidence interval; MR, Mendelian randomization; SNP, single nucleotide polymorphism.

Univariable IVW MR analyses showed that genetic instruments for insomnia was significantly associated with increased risk of AD (odds ratio [OR] = 3.02, 95% CI: 1.28 to 7.10, *P_IVW_* = 0.01) (Figure 2). No associations were observed between genetic instruments for insomnia and all‐cause dementia or VaD, or between genetic instruments for the other four sleep characteristics and any of the three dementia outcomes.

Results from MR‐Egger, WME, MR‐PRESSO outlier‐corrected, and leave‐one‐out sensitivity analyses were consistent with these findings obtained from the univariable IVW MR analysis (see Figure S2, Table S10, Figure S3). Heterogeneity was observed for MR analyses based on the results of funnel plots and Cochran's Q tests (see Figure S4 and Table S11). Although heterogeneity was observed in the funnel plots and Cochran's Q tests (see Figure S4 and Table S11), no evidence of horizontal pleiotropy in our analyses was found (see Table S12).

### 3.3 Multivariable MR analysis

**FIGURE 3:**
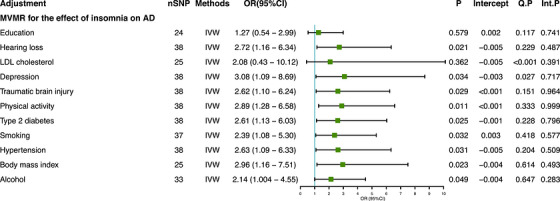
Multivariable Mendelian randomization (MVMR) analysis of insomnia and Alzheimer's disease (AD). Notes. P estimates the causal effect of insomnia on AD. A small *p*‐value (< 0.05) suggests that the estimated causal effect is statistically significant. Q.P refers to the *p*‐values from Cochran's Q test, which is used to assess heterogeneity in the MVMR analyses. A *p*‐value < 0.05 indicates evidence of heterogeneity across genetic instruments. Int.P refers to the *p*‐values derived from the Egger intercepts. MR‐Egger intercept test detects horizontal pleiotropy. A *p*‐value > 0.05 indicates no significant pleiotropy. MR‐Egger provides the most robust and widely accepted method for assessing pleiotropy while accounting for multiple correlated exposures. Other sensitivity tests, such as Mendelian Randomization Pleiotropy RESidual Sum and Outlier (MR‐PRESSO) and leave‐one‐out analysis, were not applied in the MVMR analysis because they assume a single‐exposure framework or are difficult to interpret directly in MVMR (see Ref. 36, 43–45 for details). Abbreviations. MVMR, multivariable Mendelian randomization; AD, Alzheimer's disease; OR, odds ratio; CI, confidence interval; SNP, single nucleotide polymorphism; IVW, inverse‐variance weighted.

Given the potential overlap in biological mechanisms between insomnia and other established dementia risk factors, particularly those occurring in early and midlife, we conducted MVMR analyses to estimate the direct effect of insomnia on AD while accounting for other factors highly relevant to dementia and AD. After adjusting individually for BMI, smoking, alcohol consumption, hypertension, physical activity, type 2 diabetes, hearing loss, depression, and traumatic brain injury, the IVW estimates of the effect of insomnia on AD remained statistically significant, with ORs ranging from 2.14 to 3.08 (all *P_IVW_* < 0.05; see Figure 3). However, adjustment for education attainment or LDL cholesterol markedly attenuated the association between genetically proxied insomnia and AD, leading it to become non‐significant, with ORs reduced to 1.27 (95% CI: 0.54 to 2.99, *P_IVW_* = 0.58) and 2.08 (95% CI: 0.43 to 10.12, *P_IVW_* = 0.36), respectively. Because sensitivity analyses indicated heterogeneity in the MVMR analysis of insomnia and AD, a random‐effects MVMR model was applied. MR‐Egger intercept tests showed no evidence of horizontal pleiotropy (Figure 3).

### 3.4 Additional analysis

We further investigated whether the association between insomnia and AD differed between early‐onset (<65 years) and late‐onset (≥65 years) AD. Univariable MR results indicate a stronger link between insomnia and late‐onset AD (OR = 2.97, 95% CI: 1.11 to 7.91, *P_IVW_* = 0.03), but not early‐onset AD (Figure S5). All findings were consistent when the MVMR analysis was restricted to late‐onset AD (Figure S6). Results remain the same when we included other cause‐specific dementia outcomes (Figure 2 and Table S13). Given the wide CIs observed in the univariable IVW MR analyses for VaD, additional power analyses were performed. The results showed that analyses for all‐cause dementia and AD were adequately powered across all sleep characteristics (power >0.80), whereas analyses for VaD and PDD were underpowered (Table S14). In the additional analyses exploring reverse causality between sleep characteristics and dementia, genetically predicted all‐cause dementia and AD were associated with a reduced likelihood of insomnia (Figure S7). However, after excluding *APOE* SNPs (rs769449, rs34041051, rs111371860, and rs41289514), the associations of genetic liability to all‐cause dementia, AD, and VaD with sleep characteristics were no longer statistically significant (Figure S8).

## 4 DISCUSSION

Using two‐sample MR approaches, our findings provide suggestive evidence that genetic variants associated with insomnia may increase the risk of AD. This association appears largely independent of most modifiable dementia risk factors present in earlier life, but is influenced by educational attainment and LDL cholesterol levels.

Consistent with our findings, as summarized in Table 1, previous MR studies did not provide sufficient evidence for a causal effect of daytime sleep, napping, or early rising on dementia risk.^13^, ^14^, ^15^, ^16^, ^17^, ^18^, ^19^, ^20^, ^21^, ^22^, ^23^, ^24^, ^25^, ^26^ In contrast to previous MR analyses,^15^, ^18^ our study identified a causal association between insomnia and increased AD risk. Notably, the GWAS datasets used in our study differ from those employed in earlier MR studies. Xiang et al. ^15^ used an AD GWAS comprising 954 cases and 48,733 controls drawn from 488,285 European participants, whereas our study was based on a larger AD GWAS, with 3899 cases and 214,893 controls, providing greater statistical power. In addition, the studies by Xiang et al. ^15^ and Grover et al. ^18^ used sleep‐related characteristics from the UK Biobank; however, the AD GWAS applied in these studies did not exclude UK Biobank participants, introducing a potential risk of sample overlap. By contrast, we employed a two‐sample MR design using sleep‐related characteristics from the UK Biobank and dementia outcomes from the FinnGen dataset, which eliminated sample overlap. This approach reduced weak instrument bias, minimized Type I error rates, and prevented overestimation due to the winner's curse, thereby strengthening the robustness of our findings.^47^ Although an association between insomnia and reduced cortical surface area has been reported, another study using two‐sample MR found no evidence of an association between genetically predicted insomnia and AD.^22^ The discrepancy between these findings and ours may partly reflect differences in the GWAS datasets used for AD outcomes. Specifically, that study was based on International Genomics of Alzheimer's Project (IGAP), whereas our analysis used FinnGen. IGAP relies on clinically and neuropathologically confirmed AD diagnoses, ensuring high validity. Yet, inter‐cohort variability in diagnostic procedures and case definitions may introduce heterogeneity in meta‐analytic estimates.^48^, ^49^ In comparison, FinnGen uses register‐based diagnoses within a standardized framework, which may improve consistency. Differences in the number of SNPs included in the MR analyses may reflect differences in instrument selection and underlying heterogeneity across datasets. In addition, the case–control design in IGAP may introduce misclassification of “future cases” among controls, potentially attenuating associations and biasing MR estimates toward the null, whereas longitudinal register‐based follow‐up in FinnGen with appropriate censoring may reduce this source of bias.^48^, ^49^

Insomnia may contribute to AD by increasing Aβ deposition, a key factor in cognitive decline and dementia.^50^, ^51^ Aβ accumulation is a hallmark of AD.^50^ Neuronal activity drives Aβ production, which slows during slow‐wave (deep) sleep, reducing Aβ buildup.^50^, ^51^ Insomnia, by increasing neuronal firing, boosts Aβ production and accumulation.^50^, ^51^ Slow‐wave sleep also clears metabolic waste, including Aβ,^51^, ^52^ and sleep deprivation impairs this clearance, further increasing Aβ levels. The combined effect leads to amyloid plaque formation.^51^, ^52^ Supporting this, Xie et al. showed in animals that Aβ clearance during sleep is twice as efficient as during wakefulness, with sleep deprivation raising brain Aβ levels significantly.^53^

Notably, the association between insomnia and AD was substantially attenuated after adjusting for education and LDL cholesterol in our multivariable MR analysis. Cognitive reserve theory may explain the modifying effect of education on the insomnia–AD relationship. This theory proposes that education enhances an individual's cognitive reserve, increasing their resilience to stress and reducing their susceptibility to stress‐related sleep disturbances, such as insomnia.^54^, ^55^, ^56^ The theory also suggests that individuals with high levels of education may better compensate for age‐related decline in brain function when neurodegenerative changes occur.^55^, ^57^ In addition, LDL cholesterol level may serve as a biological pathway linking insomnia to AD, or act as a co‐factor contributing to their combined effect.^58^, ^59^, ^60^ Studies have shown that chronic insomnia can lead to abnormalities in lipid metabolism, such as increased LDL cholesterol levels, and high LDL cholesterol levels have been suggested to be associated with an increased risk of AD.^58^, ^59^, ^60^ High LDL cholesterol levels may increase the risk of AD by promoting Aβ deposition, accelerating tauopathy, and impairing blood–brain barrier function.^61^ Nevertheless, the potential mediating roles of education and LDL cholesterol were not addressed in the present study and should be explored in greater depth in future research.

In this study, we did not observe evidence of a causal association between other sleep characteristics, such as sleep duration or daytime napping, and dementia risk. Consistent with our findings, a recent MR analysis supported by accelerometer estimates similarly detected no causal effect of long or short sleep duration on dementia risk.^13^ Although previous MR analyses reported that shorter sleep durations was associated with poorer fluid intelligence scores,^13^ this effect may reflect a combination of reduced slow‐wave sleep, which impairs memory encoding and consolidation, and diminished rapid eye movement sleep, which compromises attention and executive function.^13^ In the reverse MR analyses, we initially observed that genetic liability to all‐cause dementia and AD was associated with a lower likelihood of insomnia. However, these associations were attenuated and no longer statistically significant after exclusion of *APOE*‐region variants, suggesting that the observed reverse‐direction signals may have been largely driven by the *APOE* locus rather than reflecting a robust causal effect of dementia liability on sleep characteristics.

Our study has several strengths. First, taking advantage of the random assignment of alleles at conception, our MR analyses used SNPs as IVs to assess the causal relationship between self‐reported sleep characteristics and dementia. By doing so, we aimed at minimizing residual confounding and reverse causation. Second, we prioritized internal validity by using two non‐overlapping cohorts (UK Biobank and FinnGen), thereby minimizing error covariance and reducing the risk of Type I error inflation and weak‐instrument bias. Although the relatively small number of AD cases in FinnGen may reduce statistical precision, estimates from independent samples provide a more rigorous test of causality than potentially biased results from overlapping samples. However, caution is still warranted when generalizing the findings to broader European‐descent populations, given the potential for healthy volunteer bias in the UK Biobank cohort. Third, to our knowledge, this is the first time that MVMR analyses have been employed to explore the direct role of insomnia in AD development, taking into account early‐ and midlife risk factors for dementia. Fourth, we considered not only AD but also a variety of other types of dementia forms, such as VaD, to also explore a putative bidirectional causal relationship between sleep characteristics and other dementia forms. Fifth, this is the first study to explore the causal effect of insomnia on early‐onset and late‐onset AD. In addition, all participants in all GWAS datasets in this study were of European origin, helping to reduce bias due to population stratification.

At the same time, there are limitations to our current study. First, as we restricted our MR analyses to individuals of European ancestry, our findings may not be generalizable to other ancestries due to differences in allele frequencies and disease prevalence. Second, pleiotropy cannot be completely ruled out using the MR‐Egger intercept test. We used MVMR analyses to attenuate the effect of horizontal pleiotropy on the results. Third, it is often not possible in these studies to distinguish whether poor nighttime sleep or consequent daytime napping/sleepiness are associated with poor outcomes. In other words, it is not clear whether daytime napping or daytime sleepiness is a compensation for nighttime sleep deprivation or a continuation of adequate nighttime sleep. Fourth, our two‐sample MR analysis assumes a linear relationship between exposure and outcome. This approach thus may not adequately capture non‐linear associations. Fifth, the analyses examining daytime sleepiness, sleep duration, and insomnia in relation to vascular dementia, and the analyses linking all sleep characteristics to PDD appear to be underpowered (Table S14). This is largely owing to the smaller number of cases reported in previous GWAS, which may lead to an underestimation of the true relationship between sleep characteristics and these subtypes of dementia outcomes. Sixth, the sleep characteristics analyzed in the current MR study were self‐reported by participants in the original GWAS, which could lead to misclassification of sleep characteristics due to subjective assessment without a validated questionnaire. Seventh, self‐reported insomnia may be affected by memory bias, especially in older adults. Further research using more objective measures of sleep characteristics is needed. Eighth, although we conducted reverse MR analyses as additional analyses, given the limited number of SNPs in our reverse MR analysis, further investigation is needed to confirm our findings. Finally, other aspects of sleep habits need further study, for example, the predominant time period for naps and the regularity/irregularity of insomnia. Adding these aspects to future work may help refine sleep risk exposure.

The association between insomnia and AD appears to be multifaceted and indirect. Genetic variants associated with insomnia may increase the risk of AD, although this relationship appears largely attenuated by educational attainment and LDL cholesterol levels. Our findings suggest that tailored risk assessment for AD may be possible by integrating sleep patterns, lipid profiles, and sociodemographic factors. Future studies are needed to further investigate dose–response relationships between sleep characteristics, such as sleep duration, and dementia outcomes using MR approaches based on individual‐level genotype data.

## AUTHOR CONTRIBUTION

All authors participated in designing the study, generating hypotheses, interpreting the data, and critically reviewing the report. Y.G. and R.W. were primarily responsible for writing the article. Y.G. did the data analysis. All authors confirm and accept responsibility for the decision to submit for publication.

## CONFLICT OF INTEREST STATEMENT

We declare no competing interests. Author disclosures are available in the Supporting Information.

## CONSENT STATEMENT

The data used in this Mendelian randomization (MR) study were obtained from large genome‐wide association studies (GWASs), and the original cohorts included appropriate ethical clearance approvals and patient informed consent instructions. Therefore, no separate ethical approval was required for this study.

## Supporting information




**Supporting Information**: alz71592‐sup‐0001‐SupMat.xlsx

**Supporting Information**: alz71592‐sup‐0002‐ICMJE.pdf

## Data Availability

The summary‐level genome‐wide association study (GWAS) data used in this study are available from the original GWAS publications and public databases described in the manuscript. Detailed information on the data sources, including the GWAS IDs, is provided in Table S2.
